# Implications of out-of-pocket payments for the control of malaria in developing countries: A narrative review

**DOI:** 10.1097/MD.0000000000047639

**Published:** 2026-02-06

**Authors:** Emmanuel Ifeanyi Obeagu, Getrude Uzoma Obeagu

**Affiliations:** a Department of Biomedical and Laboratory Science, Africa University, Mutare, Zimbabwe; b Department of Molecular Medicine and Haematology, School of Pathology, Faculty of Health Sciences, University of the Witwatersrand, Johannesburg, South Africa; c Department of Nursing Science, School of Nursing Science, Kampala International University, Uganda.

**Keywords:** developing countries, financial barriers, health policy, healthcare access, malaria, out-of-pocket payments, socioeconomic impact

## Abstract

This narrative review investigates the implications of out-of-pocket (OOP) payments for malaria treatment in developing countries by synthesizing evidence from peer-reviewed articles, reports, and policy documents. A thematic approach was employed to analyze key areas, including economic impacts on households, barriers to accessing care, and the overall effectiveness of malaria control interventions. The review also evaluated alternative financing mechanisms and their potential to mitigate the financial burden on vulnerable populations. The findings reveal that OOP payments significantly hinder timely access to malaria treatment, disproportionately affecting low-income and rural households. Delayed treatment-seeking behavior, underutilization of preventive measures such as insecticide-treated nets, and suboptimal use of diagnostics were identified as major challenges. Furthermore, the financial burden of OOP payments exacerbates health inequities, increasing morbidity and mortality among marginalized groups. Effective alternatives, such as government subsidies, community health insurance, and donor-funded programs, were highlighted as solutions to reduce these barriers. The review concludes that reducing OOP payments is critical to improving malaria control and ensuring equitable healthcare access in developing countries. It recommends scaling up subsidized care, strengthening public health systems, and engaging in public-private partnerships to enhance the availability and affordability of malaria interventions. Policymakers are urged to prioritize financial protection strategies to achieve sustainable progress in malaria eradication efforts.

## 1. Introduction

Malaria remains a significant global health challenge, with over 247 million cases and approximately 619,000 deaths reported in 2021, according to the World Health Organization (WHO). The burden of malaria is disproportionately high in developing countries, particularly in sub-Saharan Africa, which accounts for approximately 95% of cases and 96% of deaths globally. Children under 5 years old and pregnant women are among the most vulnerable groups, suffering the highest mortality rates. Despite substantial investments in malaria control programs, the reliance on OOP payments for diagnosis, treatment, and preventive measures continues to impede progress in combating the disease.^[1–3]^ Empirical evidence highlights that OOP payments are a major barrier to accessing timely and effective malaria care. For instance, a study conducted in Nigeria reported that over 70% of households incurred catastrophic health expenditures for malaria treatment, significantly impacting their ability to afford basic needs such as food and education. Similarly, in Uganda, households spent an average of 10% of their monthly income on malaria treatment, with low-income families bearing the highest burden. These financial constraints often lead to delayed treatment-seeking behavior, increasing the risk of severe malaria complications and mortality.^[4–6]^ The financial burden of malaria is not confined to treatment alone; preventive measures such as insecticide-treated nets and indoor residual spraying (IRS) also impose significant costs on households. Research in Kenya revealed that <50% of households could afford insecticide-treated nets without external subsidies, leaving a substantial proportion of the population unprotected. Additionally, the cost of transportation to healthcare facilities further compounds the economic challenges, especially in rural areas where access to healthcare is already limited. These barriers perpetuate a cycle of poverty and ill health, undermining efforts to control malaria.^[7–9]^

OOP payments also exacerbate health inequities, disproportionately affecting marginalized populations, including rural communities, informal workers, and ethnic minorities. A study in Ethiopia found that urban households were twice as likely to afford malaria treatment compared to rural households, highlighting the geographic and socioeconomic disparities in healthcare access. Moreover, the reliance on private healthcare providers, who often charge higher fees than public facilities, further marginalizes low-income populations. These inequities highlight the need for equitable and sustainable financing mechanisms to reduce the financial burden on vulnerable groups.^[10,11]^ Drug resistance and diagnostic challenges are additional consequences of OOP payments. Limited access to affordable diagnostic services often leads to presumptive treatment, resulting in the misuse of antimalarial drugs and contributing to the emergence of drug-resistant malaria strains. A study conducted in Southeast Asia reported that 40% of malaria cases treated in private facilities were not laboratory-confirmed, increasing the risk of drug resistance and mismanagement of non-malarial febrile illnesses. This underscores the importance of integrating subsidized diagnostic services into malaria control programs to improve treatment accuracy and reduce resistance.^[12]^ Despite the challenges, evidence suggests that alternative financing mechanisms can mitigate the adverse effects of OOP payments on malaria control. For example, government-subsidized ITN distribution programs in Tanzania significantly increased ITN ownership and usage, reducing malaria prevalence by 50% in targeted regions. Similarly, Community-Based Health Insurance (CBHI) schemes in Rwanda improved access to malaria treatment, particularly among low-income households, demonstrating the potential of innovative financing models in addressing healthcare disparities.^[13,14]^ This review aims to synthesize existing evidence on the implications of OOP payments for malaria control in developing countries.

## 2. Aim

The aim of this paper is to explore the implications of OOP payments for the control of malaria in developing countries. Specifically, it seeks to investigate how out-of-pocket payments affect access to essential malaria interventions, influence health-seeking behavior, exacerbate economic burden, and hinder progress towards effective malaria control. Through this exploration, the paper aims to provide insights that can inform the development of strategies and policies aimed at mitigating the adverse effects of out-of-pocket payments and promoting equitable access to malaria prevention and treatment services.

### 2.1. Review methods

This review was conducted using a narrative approach, aimed at synthesizing current knowledge on the implications of OOP payments for malaria control in developing countries. Although narrative reviews are inherently more flexible than systematic reviews, we adopted several systematic elements – including defined search strategies, eligibility criteria, and thematic analysis – to enhance methodological transparency and replicability.

#### 2.1.1. Search strategy and data sources

A comprehensive literature search was conducted across the following databases: PubMed, Scopus, Web of Science, and the WHO Global Index Medicus. The search covered articles published between January 2010 and April 2025, to capture both foundational research and recent developments, particularly those emerging in the context of COVID-19 and its economic aftermath.

The following key search terms and Boolean combinations were used:

- “malaria” AND “out-of-pocket payments”
- “malaria control” AND “financial barriers”
- “healthcare access” AND “malaria” AND “user fees”
- “malaria” AND “CBHI”
- “malaria treatment” AND “economic burden” AND (“developing countries” OR “low- and middle-income countries” OR “LMICs”)
- “malaria” AND “informal healthcare” OR “private providers”

Where available, Medical Subject Headings (MeSH) and database-specific indexing terms were utilized to refine the search.

#### 2.1.2. *Inclusion and exclusion criteria*

##### 2.1.2.1. *Inclusion criteria*

- Peer-reviewed articles, reviews, and gray literature (e.g., reports from WHO, World Bank, Global Fund)
- Studies conducted in developing countries or LMICs as classified by the World Bank
- Studies discussing OOP payments, malaria care financing, user fees, or financial protection interventions
- English-language publications
- Articles published between January 2010 and April 2025

##### 2.1.2.2. Exclusion criteria

- Studies not related to malaria or healthcare financing
- Articles focused solely on high-income countries
- Non-English-language publications
- Commentaries or editorials without empirical or conceptual contributions

#### 2.1.3. Data extraction and thematic organization

Relevant data were extracted manually and organized under major themes, including: economic burden of OOP payments, impacts on treatment-seeking behavior, financing mechanisms (e.g., subsidies, insurance), role of informal healthcare providers, and policy implications. Where conflicting evidence emerged, it was critically appraised and incorporated into the analysis. This methodological approach allowed for an inclusive yet focused synthesis of evidence while maintaining the narrative flexibility necessary to address a broad, complex topic spanning multiple disciplines.

## 3. Ethical approval

Not applicable

## 4. Impact on access to malaria treatment: out-of-pocket payments

Out-of-pocket payments for malaria treatment have profound implications for access to healthcare services in the context of malaria control, particularly in developing countries. While malaria is a treatable disease when detected and addressed promptly, out-of-pocket payments can act as a substantial barrier, deterring individuals and communities from accessing necessary treatment.^[14,15]^ The financial burden of out-of-pocket payments often leads to delays in seeking diagnosis and treatment. When individuals or families are unable to afford the cost of diagnostic tests and medications, they may postpone visiting healthcare facilities. This delay can result in malaria progressing to more severe stages, increasing the risk of complications and mortality.^[16,17]^

High out-of-pocket costs can discourage individuals from seeking professional medical care altogether. Many may turn to self-medication or traditional remedies, which are often ineffective and may even exacerbate the disease. This avoidance of formal healthcare settings contributes to the persistence of malaria transmission.^[18,19]^ Individuals who do seek treatment but are unable to afford the full course of prescribed medications may take insufficient doses. This incomplete treatment can lead to the development of drug-resistant malaria strains, making it even more challenging to control the disease in the long term.^[20,21]^ The financial strain of out-of-pocket payments for malaria treatment can result in catastrophic healthcare expenditures for individuals and families. In many cases, the costs of treatment can deplete savings, force the sale of assets, or drive households into poverty. This, in turn, perpetuates a cycle of vulnerability and increases the risk of recurrent malaria infections.^[22]^ Vulnerable populations, such as those living in poverty, children, pregnant women, and marginalized communities, are disproportionately affected by out-of-pocket payments. These groups often bear the brunt of the financial burden, further exacerbating health inequities.^[23]^ The financial constraints imposed by out-of-pocket payments for treatment can also limit individuals’ ability to invest in preventive measures, such as insecticide-treated bed nets or IRS. This lack of preventive measures perpetuates the cycle of malaria transmission.^[24]^ Out-of-pocket payments for malaria treatment pose a significant hindrance to access to healthcare services, preventing timely diagnosis and appropriate treatment. The impact of out-of-pocket payments on access to malaria treatment can be particularly severe in developing countries where government-funded healthcare systems are limited or nonexistent. Despite being one of the countries with the highest burden of malaria globally, access to government-funded malaria treatment in Nigeria is often limited, especially in rural areas. Many individuals and families have to rely on out-of-pocket payments to access essential malaria diagnosis and treatment services. In the Democratic Republic of the Congo (DRC), where malaria is endemic and contributes significantly to the country’s disease burden, government-funded healthcare services are often inadequate. As a result, individuals frequently face financial barriers when seeking malaria treatment, resorting to out-of-pocket payments to access care. Myanmar, formerly known as Burma, is another developing country where government-funded healthcare services, including malaria treatment, may not be readily available to all citizens, particularly those in remote or conflict-affected areas. In such areas, out-of-pocket payments are common for accessing malaria diagnosis and treatment services. In Afghanistan, where malaria is endemic in certain regions, the healthcare infrastructure is fragile, and government-funded healthcare services may not reach all segments of the population. This situation leads many individuals to pay out of pocket for malaria treatment, which can be a significant financial burden for low-income households.^[25]^

Nigeria bears a significant burden of malaria, with an estimated 25% of the global malaria cases occurring in the country. High malaria prevalence rates contribute to substantial healthcare expenditures, including OOP payments, as many individuals seek treatment from private healthcare providers due to limitations in government-funded services. OOP payments for malaria treatment in Nigeria can exacerbate poverty and lead to financial hardship for affected individuals and households, particularly those living below the poverty line. Malaria is endemic throughout the DRC, with high-transmission rates and a considerable disease burden. Limited availability and accessibility of government-funded healthcare services result in individuals often resorting to OOP payments to access malaria diagnosis and treatment. The financial burden of OOP payments for malaria care can have adverse effects on healthcare-seeking behavior, leading to delays in seeking treatment or inadequate treatment due to cost constraints. Myanmar has a significant malaria burden, particularly in rural and remote areas. Despite efforts to improve healthcare infrastructure and access to government-funded services, many individuals still rely on OOP payments for malaria treatment, especially in areas with limited healthcare facilities. OOP payments for malaria care can represent a substantial portion of household expenditures, impacting the ability of households to meet other essential needs such as food, education, and housing. Malaria is endemic in certain regions of Afghanistan, with varying transmission rates across the country. Weak healthcare infrastructure and limited availability of government-funded services contribute to individuals often paying out of pocket for malaria treatment. OOP payments for malaria care can pose a significant financial burden on vulnerable populations in Afghanistan, further exacerbating poverty and hindering access to essential healthcare services.^[25]^ To overcome this challenge and enhance malaria control in developing countries, it is imperative to implement policy interventions that reduce the financial barriers faced by individuals and communities. Addressing the issue of out-of-pocket payments is a crucial step in the broader effort to alleviate the global burden of malaria.

## 5. Out-of-pocket payments for the control of malaria: effects on preventive measures

Malaria control strategies heavily rely on preventive measures, such as the distribution of insecticide-treated bed nets, IRS, and prophylactic medications. These interventions play a critical role in reducing the transmission of the disease. However, the financial burden imposed by out-of-pocket payments for malaria-related healthcare services can significantly impact the utilization and effectiveness of these preventive measures.^[26,27]^ Insecticide-treated bed nets are a cornerstone of malaria prevention, particularly in endemic regions. However, their cost can be prohibitive for many individuals and families in developing countries. Out-of-pocket payments for bed nets can deter people from purchasing them, leaving households vulnerable to malaria-carrying mosquitoes.^[28]^ IRS, another key preventive measure, involves the application of insecticides to the interior walls of homes. The cost of this intervention, when passed on to individuals or households, can discourage its adoption. As a result, fewer households benefit from this effective preventive measure.^[29]^ Preventive medications and vaccines can be essential tools in high-transmission areas. However, the out-of-pocket expenses associated with these interventions can deter individuals from seeking or completing prophylactic courses. This reduces the coverage and effectiveness of these measures.^[30]^ Vulnerable populations, such as pregnant women and children, are at increased risk of severe malaria. However, the financial burden of out-of-pocket payments may disproportionately affect these groups, making it challenging for them to access and utilize preventive measures, which are crucial for their protection.^[31]^ When individuals or communities face financial constraints due to out-of-pocket payments for malaria treatment, they may prioritize treatment over prevention. This shift in priorities can lead to a reduction in the consistent use of bed nets or compliance with other preventive practices, ultimately compromising their effectiveness.^[32^ The reduced utilization of preventive measures due to out-of-pocket payments can perpetuate the cycle of malaria transmission. This not only hinders efforts to reduce the incidence of the disease but also makes it more challenging to reach the long-term goal of malaria elimination.^[33]^ Out-of-pocket payments for malaria-related healthcare services can undermine the utilization and effectiveness of preventive measures, hindering progress in malaria control. To address this issue, it is crucial to implement policies and interventions that reduce the financial barriers faced by individuals and communities in accessing these preventive tools. Ensuring affordable and equitable access to preventive measures is essential for achieving effective malaria control in developing countries and moving closer to the goal of eradicating this devastating disease.^[34]^

## 6. Strain on healthcare systems caused by out-of-pocket payments

The burden of out-of-pocket payments for healthcare services related to malaria and other illnesses can exert significant strain on healthcare systems in developing countries. These systems are often already grappling with resource limitations and high patient loads.^[35]^ When individuals are unable to afford out-of-pocket payments for malaria treatment and related services, healthcare facilities experience reduced revenue. This decline in income can compromise the ability of healthcare institutions to maintain and improve infrastructure, provide quality care, and retain skilled healthcare personnel.^[36]^ In response to the financial barriers created by out-of-pocket payments, individuals may seek care at public health facilities, which are generally more affordable. This increased demand for services can lead to overcrowding and extended waiting times, reducing the quality of care delivered and deterring patients from seeking treatment.^[37]^ Healthcare facilities may face resource allocation dilemmas as they try to balance limited resources among the growing demands of patients who need malaria treatment. This can lead to shortages of critical supplies, such as antimalarial drugs, diagnostic tests, and trained healthcare workers, further impeding the quality of care provided.^[38]^ The strain imposed by out-of-pocket payments can place additional pressure on healthcare workers, who may have to handle larger caseloads with fewer resources. Overworked and understaffed healthcare facilities can lead to burnout among healthcare providers, affecting the quality of patient care.^[39]^ Out-of-pocket payments can exacerbate healthcare inequities by limiting access to those who cannot afford the costs. This results in a 2-tiered healthcare system, where individuals with financial means receive better care while those who lack resources are left with suboptimal or no treatment.^[40]^ Inadequate revenue due to reduced out-of-pocket payments can make it difficult for healthcare facilities to maintain and upgrade their infrastructure. This can lead to crumbling facilities and outdated equipment, which negatively impact patient care.^[41]^ In times of malaria outbreaks or epidemics, the strain on healthcare systems caused by out-of-pocket payments can hinder a rapid and effective response. The surge in cases can overwhelm facilities that are already struggling with insufficient resources and finances.^[42]^ Out-of-pocket payments for healthcare services, including malaria treatment, place significant strain on already fragile healthcare systems in developing countries. Addressing this issue is not only essential for improving the quality of care and patient outcomes but also for achieving broader health equity and successful malaria control efforts. Policy interventions and financing mechanisms that alleviate the financial burden on individuals while ensuring sustainable funding for healthcare systems are essential steps toward enhancing the resilience and capacity of healthcare infrastructure in these regions.^[43]^

## 7. Policy interventions to mitigate the implications of out-of-pocket payments on malaria control

To address the adverse effects of out-of-pocket payments on malaria control in developing countries, a range of policy interventions can be implemented.^[44]^ Implement and expand UHC programs to provide access to a comprehensive package of essential healthcare services, including malaria prevention, diagnosis, and treatment, without requiring out-of-pocket payments.^[45]^ Ensure that vulnerable and marginalized populations are specifically targeted and covered by UHC initiatives. Subsidize the cost of antimalarial drugs and diagnostic tests to make them more affordable for individuals. This can involve government subsidies, international aid, or partnerships with pharmaceutical companies to lower the cost of essential medications and tests.^[46]^ Promote the establishment of CBHI schemes, where individuals and households contribute to a shared fund that covers healthcare expenses, including those related to malaria.^[47]^ Ensure that the premium contributions are affordable and consider income-based scales to reduce financial barriers for low-income populations. Invest in healthcare infrastructure by building and maintaining healthcare facilities and ensuring the availability of trained healthcare workers in malaria-endemic areas.^[48]^ Allocate resources to maintain and upgrade healthcare infrastructure, including diagnostic laboratories and treatment centers. Remove user fees for malaria-related services, including diagnosis and treatment, particularly in public healthcare facilities. This policy eliminates direct out-of-pocket payments at the point of service.^[49]^ Compensate for lost revenue through alternative financing mechanisms, such as government funding or international aid. Launch public awareness campaigns to educate communities about the importance of early diagnosis and treatment for malaria and the effective use of preventive measures.^[50]^ Empower individuals to recognize the signs and symptoms of malaria and seek timely care, emphasizing that such care should be affordable and accessible. Integrate malaria control efforts with other health programs, such as maternal and child health services, to improve the overall effectiveness of healthcare delivery.^[51]^ Implement a holistic approach to healthcare that addresses multiple health needs simultaneously. Establish robust monitoring and evaluation systems to track the impact of policy interventions on reducing out-of-pocket payments and improving malaria control.^[52]^ Use data and evidence to make informed policy adjustments and improvements. Collaborate with international organizations, NGOs, and donor agencies to secure financial and technical support for malaria control programs.^[53]^ Leverage partnerships to ensure the sustainability of policy interventions and to expand their reach. Addressing the implications of OOP payments on malaria control in developing countries requires a multifaceted approach. These policy interventions not only reduce the financial burden on individuals but also enhance the overall effectiveness of malaria control efforts. By implementing these policies and fostering collaboration between governments, international organizations, and local communities, we can significantly improve access to malaria services and make substantial progress toward the goal of malaria elimination in these vulnerable regions.^[54,55]^

## 8. Impact of out-of-pocket payments on malaria control efforts in developing countries

The review of the 11 key studies and reports highlighted the widespread and multifaceted impact of OOP payments on malaria control efforts in developing countries, particularly focusing on sub-Saharan Africa, South Asia, and parts of Latin America. These regions bear the highest burden of malaria, with millions of cases and thousands of deaths annually.

## 9. Accessibility and healthcare-seeking behavior

The studies consistently indicated that high OOP costs significantly impede access to malaria treatment and prevention services. In countries like Nigeria, Kenya, and India, where the prevalence of malaria is high, the direct costs of treatment, including consultation fees, diagnostic tests, and medications, deter many from seeking timely care. For instance, a study in Kenya revealed that up to 40% of households delayed or avoided seeking formal healthcare due to financial constraints, opting instead for self-medication or traditional remedies. This delay in seeking appropriate treatment often results in severe health outcomes and increased transmission rates within communities.^[56]^

## 10. Financial burden on households

The economic impact on households was profound across all reviewed regions. In Ethiopia and Uganda, for example, households were reported to spend between 5% and 25% of their annual income on malaria treatment and prevention. Such expenditures are catastrophic for low-income families, often forcing them to make difficult choices between healthcare and other essential needs like food, education, and housing. The reviewed studies also highlighted that many families resort to borrowing money or selling assets to cover the costs, pushing them deeper into poverty. In India, it was found that households in rural areas, in particular, are disproportionately affected due to their lower income levels and less access to subsidized healthcare services.^[57]^

## 11. Public health outcomes

The broader public health implications of OOP payments were stark. In regions like Tanzania and Ghana, high OOP costs were correlated with higher malaria prevalence and mortality rates. The lack of affordable healthcare options undermines public health efforts to control and eliminate malaria. The reviewed studies pointed out that insufficient access to affordable diagnostic and treatment services leads to the misuse of antimalarial drugs, contributing to the growing issue of drug resistance. For example, in Cambodia, the high cost of accurate diagnostic tests led to overreliance on presumptive treatment, exacerbating the problem of drug resistance.^[57]^

## 12. Policy implications and recommendations

The review also underscored the necessity of policy interventions to mitigate the impact of OOP payments. Successful examples were noted in Rwanda and Thailand, where government-subsidized healthcare programs and CBHI schemes significantly improved access to malaria treatment and reduced the financial burden on households. These models demonstrate the potential benefits of similar approaches in other malaria-endemic countries.^[58]^

## 13. Conflicting evidence in malaria financing and access to care

Despite a general consensus on the detrimental effects of OOP payments in malaria-endemic regions, the literature presents conflicting findings regarding the effectiveness of specific financial interventions and healthcare delivery models.

### 13.1. Community-based health insurance: promise versus practical challenges

CBHI schemes have been proposed as an equitable mechanism to reduce OOP expenditures and improve access to malaria care. Several studies highlight their benefits in increasing healthcare utilization, reducing financial hardship, and promoting early treatment-seeking behavior, particularly in rural populations.^[59]^ However, contrasting evidence points to challenges related to sustainability, low enrollment rates, and adverse selection. In some cases, the poorest households – who would benefit most – are least likely to enroll due to affordability barriers or limited awareness.^[60]^ Moreover, variability in the quality of services under CBHI programs has led to skepticism about their scalability and long-term viability in fragile health systems.

### 13.2. Informal healthcare providers: a double-edged sword

In many malaria-endemic settings, informal drug vendors and traditional healers serve as the first point of contact for febrile illnesses. On 1 hand, these providers offer immediate, geographically accessible, and often culturally accepted services, especially where public health facilities are absent or under-resourced.^[61]^ On the other hand, concerns persist regarding misdiagnosis, inappropriate use of antimalarials, and lack of adherence to national treatment guidelines. Some studies argue that these providers fill critical gaps in care; others caution that reliance on the informal sector may delay appropriate treatment and undermine national malaria surveillance and control programs.^[62,63]^

### 13.3. User-fee abolition: effective or overestimated?

The removal of user fees for malaria diagnostics and treatment has been widely promoted to reduce financial barriers. Evidence from several countries, including Zambia, Uganda, and Senegal, suggests that eliminating user fees improves service utilization and reduces mortality, particularly among children under 5. Nonetheless, other studies report mixed outcomes, with fee abolition sometimes leading to overburdened health systems, drug stock-outs, and deterioration in service quality. In the absence of compensatory funding or adequate resource planning, user-fee removal may create unintended negative consequences that compromise patient outcomes and staff morale.^[63,64]^

## 14. Discussion

The review of the implications of OOP payments for malaria control in developing countries reveals critical insights into how financial barriers affect health outcomes, healthcare accessibility, and overall public health efforts. The studies spanned several malaria-endemic regions, including sub-Saharan Africa (Nigeria, Kenya, Ethiopia, Uganda, Tanzania, Ghana), South Asia (India), Southeast Asia (Cambodia), and parts of Latin America. These diverse contexts highlight the widespread and systemic nature of the issues related to OOP payments.^[56]^ The deterrent effect of high OOP costs on healthcare-seeking behavior is a significant concern across all reviewed regions. In Nigeria and Kenya, for example, the financial burden of malaria treatment leads many households to delay or forgo professional medical care, opting instead for self-medication or traditional healers. This delay in accessing appropriate care not only worsens health outcomes for individuals but also contributes to the continued spread of malaria within communities. The situation in India mirrors this trend, particularly in rural areas where lower income levels exacerbate the impact of high treatment costs. The reluctance to seek timely medical intervention due to cost concerns is a common thread that hinders effective malaria control across these countries.^[57]^

The economic strain imposed by OOP payments is profound and often catastrophic for households in malaria-endemic regions. In Ethiopia and Uganda, the percentage of annual household income spent on malaria treatment can range from 5% to 25%. This substantial financial burden forces families to make tough choices, often at the expense of other critical needs such as food, education, and housing. The review highlighted that in countries like Tanzania and Ghana, the need to borrow money or sell assets to cover healthcare costs further entrenches families in poverty. This cycle of economic hardship and poor health outcomes creates a feedback loop that is difficult to break, undermining both household stability and broader economic development.^[58]^ The broader public health implications of high OOP payments are significant. In countries such as Tanzania and Ghana, the correlation between high healthcare costs and elevated malaria prevalence and mortality rates is evident. The inability to afford proper diagnostic and treatment services leads to the overuse and misuse of antimalarial drugs, contributing to the alarming rise in drug-resistant malaria strains. The situation in Cambodia, where high diagnostic costs result in reliance on presumptive treatment, underscores the global challenge of maintaining effective malaria control in the face of financial barriers. These issues highlight the urgent need for more accessible and affordable healthcare solutions to prevent the deterioration of public health and the spread of resistant malaria strains.^[56]^

Addressing the financial barriers posed by OOP payments requires comprehensive and multifaceted policy interventions. Successful examples from Rwanda and Thailand illustrate the potential benefits of government-subsidized healthcare programs and CBHI schemes. These initiatives have significantly improved access to malaria treatment and reduced the financial burden on households, demonstrating a viable path forward for other malaria-endemic countries.^[57]^ Expanding health insurance coverage and subsidizing malaria treatment and prevention measures are critical steps that governments and international organizations should prioritize. Strengthening healthcare systems, including training healthcare workers, improving supply chains, and enhancing healthcare infrastructure, is equally important. Public-private partnerships can also play a vital role in mobilizing resources and ensuring the distribution of affordable or free malaria treatments and preventive tools.^[58]^

## 15. Limitations of the review

This review has several limitations that should be acknowledged. First, as a narrative review, the methodology does not follow the strict systematic protocols used in systematic reviews or meta-analyses. While efforts were made to include diverse and relevant sources, the selection and interpretation of literature may be subject to subjective bias, potentially influencing the scope and balance of evidence presented. Second, publication bias may have influenced the available evidence base. Studies reporting significant or positive findings – especially regarding health financing interventions – are more likely to be published and indexed, while negative or inconclusive results may be underrepresented. Third, regional disparities in data availability present a challenge. Much of the literature on out-of-pocket payments and malaria control is concentrated in sub-Saharan Africa, with fewer studies from Southeast Asia, Latin America, or conflict-affected regions. As a result, the generalizability of findings across all developing countries may be limited. Finally, the review excluded non-English-language publications, which may have resulted in the omission of relevant studies, particularly from Francophone and Lusophone countries in Africa, as well as regions in Asia and Latin America where significant research may be published in local languages.

## 16. Conclusion

Out-of-pocket payments continue to pose significant barriers to timely diagnosis, effective treatment, and overall malaria control in developing countries. As this review has demonstrated, financial hardship not only limits access to life-saving care but also deepens health inequities, especially among vulnerable populations such as children under 5, pregnant women, and the rural poor. To move toward equitable and sustainable malaria control, several actionable strategies emerge from the current body of evidence. The implementation of targeted subsidies for malaria diagnostics and treatment – particularly for high-risk and low-income groups – can significantly reduce financial strain and encourage early treatment-seeking behavior. These subsidies must be strategically designed to avoid inefficiencies and ensure that benefits reach those most in need.

The strengthening of risk-pooling mechanisms, such as CBHI and national health financing schemes, offers a pathway to reduce reliance on out-of-pocket spending. However, for these mechanisms to be effective, they must be adequately funded, equitably governed, and aligned with broader health system reforms. Innovations such as mobile-based premium payments and flexible enrollment policies can enhance accessibility and uptake, especially in remote areas. There is a pressing need for improved accountability and transparency in donor-financed malaria interventions. While international funding remains critical in supporting malaria programs, evidence indicates that fragmented financing, weak oversight, and misalignment with national priorities can undermine long-term impact. Strengthening monitoring frameworks, involving local stakeholders in decision-making, and ensuring regular audit and public reporting can enhance the effectiveness and sustainability of external aid.

## Author contributions

**Conceptualization:** Emmanuel Ifeanyi Obeagu.

**Methodology:** Emmanuel Ifeanyi Obeagu, Getrude Uzoma Obeagu.

**Supervision:** Emmanuel Ifeanyi Obeagu.

**Validation:** Emmanuel Ifeanyi Obeagu.

**Visualization:** Emmanuel Ifeanyi Obeagu.

**Writing** – **original draft:** Emmanuel Ifeanyi Obeagu, Getrude Uzoma Obeagu.

**Writing** – **review & editing:** Emmanuel Ifeanyi Obeagu, Getrude Uzoma Obeagu.
